# DNA Yield and Degradation in Skeletal Remains from Two Slovenian Second World War Mass Graves: A Comparative Study of Different Bone Types

**DOI:** 10.3390/genes17060719

**Published:** 2026-06-21

**Authors:** Irena Zupanič Pajnič, Tomaž Zupanc, Eva Podovšovnik

**Affiliations:** 1 Institute of Forensic Medicine, Faculty of Medicine, University of Ljubljana, Korytkova 2, 1000 Ljubljana, Slovenia; 2 Orthopedic Hospital of Valdoltra, Jadranska Cesta 31, 6280 Ankaran, Slovenia

**Keywords:** skeletal remains, Second World War, mass graves, human identification, DNA quantification, DNA degradation

## Abstract

**Background:** The genetic identification of Second World War (WWII) victims in Slovenia is a significant forensic challenge due to the varying taphonomic conditions of mass graves and the high degradation of skeletal remains. While recent studies highlight the potential of small cancellous bones and petrous parts, the variability of DNA preservation across different mass grave contexts remains under-investigated. **Objectives:** This study aimed to compare DNA quantity and quality across different skeletal elements grouped by anatomical and structural characteristics, specifically evaluating how two distinct burial sites—Konfin II and Huda Jama—influenced DNA preservation. **Materials and Methods:** A complete dataset of 785 samples was analyzed, integrating 114 newly processed samples from Huda Jama with previously published data from both sites. DNA was extracted using a total demineralization protocol and purified via the Biorobot EZ1 system. Quantification and degradation assessment were performed using the PowerQuant qPCR kit. Skeletal elements were categorized into six groups: temporal—pars petrosa, big long bones, torso bones, small long bones, short/sesamoid bones, and teeth. **Results:** Statistical analysis revealed significant differences in DNA yield and degradation between the two sites. Huda Jama samples exhibited significantly higher DNA yields in small long bones and short/sesamoid bones compared to Konfin II. Conversely, Konfin II showed superior DNA yield in teeth and torso bones. Regarding DNA quality, teeth were significantly more degraded in Huda Jama, while big long bones showed significantly higher degradation in Konfin II. No significant differences in the degradation index were observed between the sites for other skeletal elements, including small long bones and short/sesamoid bones. The temporal—pars petrosa remained a high-performing element at both locations. **Conclusions:** DNA preservation is highly site-specific and influenced by the complex taphonomic conditions of the burial site. While small cancellous bones are excellent candidates for DNA recovery in some environments (Huda Jama), teeth and torso bones may provide higher yields in others (Konfin II). However, the rate of DNA fragmentation (degradation) varies independently of yield, as seen in the extreme degradation of teeth in Huda Jama. A multi-sample strategy, prioritizing the petrous bone while accounting for site-specific preservation patterns, is essential for maximizing identification success in highly degraded skeletal remains from WWII mass graves in Slovenia.

## 1. Introduction

The systematic identification of victims from Second World War (WWII) mass graves in Slovenia represents a profound forensic and humanitarian challenge. The primary goal of these investigations is the genetic identification of remains to return them to their families, a process that relies heavily on the successful recovery of nuclear DNA [1,2]. However, skeletal remains found in mass graves are often exposed to harsh taphonomic conditions, leading to severe DNA fragmentation and low template amounts, which complicate the production of useful genetic profiles [3,4]. After death, DNA undergoes progressive degradation, including fragmentation and chemical modifications such as deamination, which are heavily influenced by the surrounding geochemical environment [3,5].

Studies conducted on remains from sites like the Konfin II abyss have shown that DNA preservation is highly dependent on the type of skeletal element analyzed [6]. Traditionally, forensic protocols preferred dense cortical bones, such as the femur, and teeth for analysis [7,8]. Recent studies have shifted focus towards alternative skeletal elements that might offer better DNA protection under adverse conditions, such as the petrous bone, tooth cementum, and specific small bones of the hands and feet [9,10,11]. This is attributed to the protective micro-environments provided by the specific density and structure of these bones, as DNA preservation has been shown to differ significantly between compact and trabecular bone [12]. In particular, the petrous part has been identified as a superior DNA reservoir in several recent bioarchaeological and forensic studies due to the extreme mineral density of the otic capsule [13].

The preservation of DNA in skeletal remains is a complex process influenced by various taphonomic factors, including humidity, soil pH, and temperature, which collectively dictate the rate of DNA decay [14,15]. While these factors are known to drive different diagenetic outcomes in environments such as mine shafts or karst abyss settings [16,17], their precise longitudinal impact is often difficult to quantify in historical mass grave contexts. Due to the lack of specific environmental monitoring data for many WWII sites, a comparative approach focusing on the observed differences in DNA yield and degradation across various skeletal elements remains a crucial proxy for understanding site-specific preservation patterns [5,8].

This study compares DNA quantity and quality from 785 skeletal elements recovered from the Konfin II and Huda Jama sites. By grouping skeletal remains into six distinct categories—temporal—pars petrosa, big long bones, torso bones, small long bones, short/sesamoid bones, and teeth—we evaluate which elements are most resilient to degradation in different Slovenian burial contexts. The findings aim to refine sampling protocols and improve the efficiency of DNA-based identifications in future forensic cases involving highly degraded remains [18].

## 2. Materials and Methods

### 2.1. Description of Konfin II and Huda Jama Mass Grave and Sample Selection

The study included a total of 785 skeletal samples recovered from two distinct WWII mass grave sites in Slovenia, Konfin II and Huda Jama, which represented different taphonomic environments and provided a basis for comparing DNA preservation in skeletal remains. To enable a robust comparative analysis of DNA preservation across skeletal elements, we integrated previously published data from Konfin II (n = 560) and Huda Jama (n = 111) with a newly analyzed dataset of 114 samples from the Huda Jama site. All samples were collected during the forensic excavations of the mass graves and were selected to represent various anatomical and structural groups of bones.

The Konfin II mass grave was located in the Ribnica region and was a typical example of a karst abyss. This natural vertical shaft served as a site for the clandestine disposal of victims during WWII. The environmental conditions within the abyss were characterized by constant low temperatures and high humidity, typical of the Slovenian karst underground. The skeletal remains at this site were found in a chaotic state, mixed with soil, rocks, and debris that had accumulated over decades, creating a complex microenvironment in which the bones were in direct contact with moist, alkaline soil and limestone [6,19]. A total of 560 skeletal samples were collected from this location for genetic analysis.

In contrast, the Huda Jama mass grave is situated within the Barbara Pit of a coal mine near Laško. This site differed significantly from a natural abyss as it was a man-made horizontal mine shaft. To conceal the grave, the remains were placed behind several thick barriers made of concrete, bricks, and clay. This artificial isolation created a unique, almost hermetically sealed environment characterized by extreme humidity and the presence of mine gases. These specific conditions led to unusual preservation processes, including the partial mummification of some remains, which was rarely observed in other types of mass graves in the region [11]. For this research, 225 skeletal elements were sampled from the Huda Jama site.

Both sites represented significant forensic challenges due to the large number of individuals and the degree of commingling of the remains. Specifically, while the excavation at Huda Jama allowed for the recovery of skeletons in their anatomical position, the remains in the Konfin II karst abyss were commingled, further complicating the individualization process. By comparing the karst environment of Konfin II with the industrial, enclosed environment of Huda Jama, the study aimed to identify which skeletal elements are most resilient to degradation across different burial contexts.

The statistical analysis approach for this study involved consolidating DNA data from various skeletal elements previously published by our research group in peer-reviewed literature. For the Konfin II site, data for the first rib were obtained from Božič [20], while the 12th thoracic vertebra was sourced from Benedik Bevc [21]. Information regarding metacarpals and metatarsals was retrieved from Inkret [19], and data for teeth, patellae, calcaneus, medial cuneiform, cuboid, navicular, talus, petrous bone, tibia, and femur were extracted from Inkret [6]. Regarding the Huda Jama site, the study by Zupanc [11] processed all representative skeletal elements of the human body for three individual skeletons, totaling 156 samples. For the statistical analysis in the present study, we included 111 skeletal elements from Zupanc [11] that corresponded to our six established skeletal groups; these groups were described in detail in the Statistical Analysis section (Section 2.4). Additionally, for this article, we included data from 114 skeletal elements newly sampled from five additional skeletons at the Huda Jama site, including teeth, the petrous part of the temporal bone, femur, tibia, patella, first rib, lumbar and thoracic vertebrae, calcaneus, talus, navicular, cuboid, cuneiform bones, capitate, metacarpals and metatarsals I to V, and proximal and distal phalanges of the hands and feet. In total, the 225 samples from the Huda Jama site originated from eight distinct, anthropologically defined skeletons (three previously published and five newly analyzed in the present study). However, it must be explicitly mentioned that the assignment of the sampled bones to these numbered individuals was strictly based on anatomical and anthropological criteria during excavation and was not genetically verified. Similarly, for the Konfin II site, the remains were highly commingled, and the sampled bones were grouped and analyzed strictly by their anatomical and structural types rather than individual genetic profiles.

In total, the statistical comparison included 560 bones and teeth from Konfin II and 225 from Huda Jama. Based on their anatomical type and structural density, these skeletal remains were categorized into six distinct groups for the subsequent statistical analysis. A comprehensive table listing all sampled skeletal elements for the Huda Jama site is available in the Supplementary Material (SM), Table S1.

Informed consent was acquired from individuals included in the elimination database. Research received approval from the National Medical Ethics Committee of the Republic of Slovenia (102/11/2014 and 0120-22/2017/3).

### 2.2. DNA Extraction

All samples were processed in the laboratory following strict anti-contamination protocols. The bone surfaces were first mechanically cleaned to remove exogenous contaminants. Mechanical cleaning involved the removal of the outer layer, followed by chemical cleaning (soaking in 5% Alconox (Sigma-Aldrich, St Louis, MO, USA), and rinsing with sterile bi-distilled water and 80% ethanol). To further eliminate potential surface contamination, all samples were exposed to ultraviolet (UV) light (254 nm) for 30 min on each side. DNA was extracted using a total demineralization protocol. To maintain the authenticity of the ancient DNA and ensure the integrity of the results, rigorous anti-contamination measures were prioritized throughout the study. All bone and tooth preparation and extraction procedures were physically isolated from post-extraction tasks [2]. Handling of the skeletal remains took place in a dedicated MC3 microbiological safety cabinet (Iskra Pio, Šentjernej, Slovenia), equipped with HEPA filtration and integrated UV lighting (254 nm) to maintain a sterile environment. To monitor for potential environmental or reagent contamination, extraction-negative controls (ENC) were incorporated into every batch [22], and the number of samples per batch was limited to 12 to minimize the risk of cross-contamination. Furthermore, a comprehensive elimination database was established, containing genetic profiles of all personnel involved in the excavation, anthropological study, and laboratory processing. To prevent any contact between modern and ancient DNA, separate automated systems were utilized: a BioRobot EZ1 (Qiagen, Hilden, Germany) was used for elimination of database samples, while an EZ1 Advanced XL (Qiagen), reserved exclusively for degraded skeletal remains, was employed for the bone and tooth samples.

Sterilization protocols for the laboratory environment and equipment were equally stringent. All work surfaces were decontaminated with 6% sodium hypochlorite, sterile bi-distilled water, and 80% ethanol, followed by 20 min of UV radiation (254 nm) both before and after use. Similarly, all reagents and laboratory consumables, unless certified DNA-free, were autoclaved and UV-treated (254 nm) for 20 min using a BLX-Multichannel BioLink DNA Crosslinker (Vilber, Collégien, France). Grinding and cutting tools underwent a multi-step disinfection process, including chemical cleaning (subsequent washing in 6% sodium hypochlorite, sterile bi-distilled water, and 80% ethanol), autoclaving in a Europa B xp sterilizer (Tecno-Gaz, Parma, Italy) at 134 °C for 45 min, and subsequent UV exposure (254 nm).

The processing of skeletal remains from five individuals from the Huda Jama site began with thorough surface cleaning using 5% Alconox (Sigma-Aldrich), sterile bi-distilled water, and 80% ethanol. The bones were then sectioned with a sterile diamond saw (Schick, Schemmerhofen, Germany). In accordance with Pinhasi’s method [13], the highly mineralized part of the petrous bone within the otic capsule was isolated. To facilitate efficient grinding, light incisions were made on the compact bone surfaces. A critical step involved pre-cooling all skeletal elements in liquid nitrogen immediately before cutting and grinding to prevent thermal DNA degradation due to friction. Once pulverized into a fine powder using a Bead Beater MillMix 20 homogenizer (Tehtnica, Domel, Železniki, Slovenia), 0.5 g of the material was used for DNA extraction. The cleaning, pulverization, demineralization, and purification stages followed the high-efficiency protocol described by Zupanič Pajnič [2] and the manufacturer’s instructions [23].

Regarding the remaining 671 samples included from previously published studies (560 from Konfin II and 111 from Huda Jama), identical strict anti-contamination protocols, laboratory environment controls, and clean-room handling standards were applied. All samples across the integrated dataset were processed in the same specialized facility under the same precautionary measures, ensuring full consistency in material handling and minimizing the risk of inter-study experimental bias. To guarantee direct comparability when interpreting and comparing DNA yield and degradation results across the entire dataset, DNA extraction for all 785 samples followed the core total demineralization logic combined with magnetic bead-based purification on automated Qiagen platforms. Extraction steps and demineralization protocols remained uniform across all sub-studies, ensuring that the observed differences in DNA metrics are taphonomic rather than method-driven.

### 2.3. DNA Quantification

To assess DNA concentration and the extent of degradation in bone and tooth samples, quantitative real-time PCR (qPCR) was employed. Raw data processing and export were conducted using the QuantStudio 5 Real-Time PCR System and the Quant-Studio Design and Analysis Software 1.5.1 (Applied Biosystems, AB, Foster City, CA, USA). Following the manufacturer’s protocols [24], the PowerQuant System (Promega Corporation, Madison, WI, USA) was utilized for the simultaneous detection of three genomic targets: a short autosomal fragment (85 bp, Auto target) to determine nuclear DNA concentration, a Y-chromosomal fragment (Y target), and a long autosomal fragment (294 bp, Deg target). The DNA degradation index (DI) was calculated as the standard ratio of the concentration of the small autosomal target to the concentration of the large autosomal target, following the manufacturer’s technical specifications for the PowerQuant System [24].

The relationship between the Auto and Deg target values provided the basis for calculating the DNA degradation (Auto/Deg ratio). All primary metrics—including Auto, Deg, and Y values, the Auto/Deg ratio, and internal PCR control (IPC) shifts—were analyzed alongside their respective standard curves using the PowerQuant Analysis Tool (Promega). To ensure reaction reliability, the PowerQuant System (Promega) incorporates an IPC to monitor for the presence of PCR inhibitors. In adherence to the manufacturer’s guidelines [24], the thresholds for the IPC Shift and the Auto/Deg ratio were established at 0.3 and 2, respectively.

### 2.4. Statistical Analysis

A comprehensive statistical evaluation was performed to compare DNA preservation in skeletal remains from two distinct WWII mass grave sites: the Konfin II karst abyss and the Huda Jama mine shaft. Although both sites date to the same historical period, they represent different environmental micro-niches, ranging from a natural subterranean cave system to an enclosed, man-made industrial shaft. These settings provide a unique opportunity to assess how different taphonomic conditions influence the biological integrity of skeletal remains over several decades. For this analysis, two primary quantitative parameters were utilized to characterize the state of DNA preservation: the Auto target, expressed in ng DNA/μL, which serves as a measure of total human DNA quantity, and the Auto/Deg ratio, also known as the degradation index, which serves as a proxy for DNA quality and fragmentation. Both parameter values were acquired using the PowerQuant Analysis tool 4.8.0 (Promega).

To explore the effects of location and bone type on genetic material stability, the following research hypotheses emerged. Hypothesis 1 suggests that there are statistically significant differences in the amount of DNA in different groups of skeletal elements across the two locations, while Hypothesis 2 suggests that there are statistically significant differences in the degradation index by different groups of skeletal elements in these two locations. First, the normality of the distribution was tested using the Kolmogorov–Smirnov test. In order to test the research hypotheses, the 95% confidence intervals for medians were calculated using bootstrapping with 10,000 samples. All statistical analyses were performed using IBM SPSS Statistics, version 26.0. There were 560 samples originally included in the database for Konfin II and 225 samples for Huda Jama. Cases with missing values for undetermined amounts of extracted DNA were omitted from further statistical analysis, resulting in a final comparison of 528 samples for the Konfin II database and 201 samples for the Huda Jama database.

Bones were classified into six specific morphological groups. The temporal—pars petrosa group consisted of the temporal—pars petrosa. The big long bones group included the femur and tibia. The torso bones group comprised the first rib, lumbar vertebrae, and thoracic vertebrae. The small long bones group included metacarpals I to V, metatarsals I to V, and various phalanges, including the 1st proximal and distal hand phalanges and the 1st proximal and distal foot phalanges. The short and sesamoid bones group consisted of the patella, calcaneus, talus, navicular, capitate, cuboid, and the intermediate, lateral, and medial cuneiforms. Finally, the teeth group included mandibular and maxillary incisors, canines, premolars, and molars. Missing values for undetermined results in the degradation index were computed using a formula:

$$Value\left(i\right)=\;Max\left(i\right)+SD(i)$$

where i stands for separate groups of skeletal elements, max(i) stands for the maximum value in a specific group, and SD(i) stands for the standard deviation for a specific group of bones (For the group of temporal—pars petrosa bones found in Huda Jama, the maximum value is 17.99 and the standard deviation is 4.83. As such, a value of 17.99 + 4.83 (resulting in 22.82) was input in the case of the missing value for temporal—pars petrosa bones found in Huda Jama. In Table 1, descriptive statistics for bone groups in Huda Jama are presented.

The highest amount (98.01) of the Auto/Deg ratio in Huda Jama was found in small long bones, followed by teeth (47.6), short and sesamoid bones (34.53), torso bones (25.91), temporal—pars petrosa (17.99), and big long bones (8.25).

In Table 2, descriptive statistics for bone groups in KonfinII are presented.

The highest amount (444.73) of the Auto/Deg ratio in KonfinII was found in teeth, followed by metacarpals (198.59), big long bones (100.35), metatarsals (78.35), torso bones (71.18), temporal—pars petrosa (64.85), and short and sesamoid bones (53.93). In the following, metatarsals and metacarpals were grouped as small long bones.

## 3. Results

### 3.1. DNA Quantification

The data generated by the PowerQuant System (Promega), encompassing various bone and tooth sample properties for the Huda Jama site alongside DNA quantity and quality metrics—such as the Auto, Deg, and Y targets, IPC Shift, and the Auto/Deg ratio—are detailed in the Supplementary Materials. To ensure high precision and reduce experimental variability, every amplification was carried out in duplicate. These duplicate averages then served as the basis for all subsequent statistical calculations. Concentrations for the Auto, Deg, and Y targets are reported in units of ng DNA/µL of extract (see Supplementary Materials, Table S1). Samples with missing values for undetermined amounts of extracted DNA were omitted from further statistical analysis, 32 from the Konfin II, and 24 from the Huda Jama site.

The efficiency of the purification process using the EZ1&2 DNA Investigator kit (Qiagen) magnetic bead technology was confirmed by the fact that nearly all samples exhibited IPC values below the 0.3 threshold. Inhibitor presence was identified in only a single instance—an intermediate cuneiform from skeleton C—where the IPC Shift reached 0.73, exceeding the 0.3 limit (see Supplementary Materials, Table S1, highlighted in red).

DNA degradation varied significantly between the two sites only for specific skeletal elements. In Huda Jama, the highest fragmentation was observed in the teeth group, with a median Auto/Deg ratio of 64.16, which was significantly higher than in Konfin II (13.42 < Me < 25.79). Conversely, big long bones exhibited significantly higher degradation in Konfin II (11.49 < Me < 32.97) than in Huda Jama (3.32 < Me < 5.18). For all other skeletal groups, including small long bones and short/sesamoid bones, no statistically significant differences in the degradation index were observed between the two sites, indicating that while DNA yield differed, the degree of fragmentation remained comparable for these elements. Regarding the extraction-negative controls (ENC), PowerQuant targets remained undetected in the majority of cases. In the few instances where amplification in qPCR occurred, DNA concentrations did not surpass the validated detection limit. Based on the developmental validation conducted by Ewing [25], a minimum concentration of 0.5 pg of DNA per μl of extract is required for dependable quantification with the PowerQuant qPCR kit (Promega); this value was therefore established as our detection limit for ENCs, confirming that no significant contamination issues occurred.

### 3.2. Statistical Analysis

#### 3.2.1. Results for Huda Jama

Initially, descriptive statistics (after imputation of missing values, Table 3) were calculated, and the normality of distribution was assessed for the DNA concentration (Auto target [ng DNA/μL]) and the degradation index (Auto/Deg ratio) for the Huda Jama skeletal remains (Table 4).

The highest median for the AutoDeg ratio of bones found in Huda Jama was detected in teeth (64,16). The other groups of bones had a median lower than 12.

The One-Sample Kolmogorov–Smirnov test revealed that the distribution for both variables significantly deviated from normality (*p* < 0.001 in both cases). Consequently, non-parametric tests were employed for all subsequent hypothesis testing.

**Figure 1:**
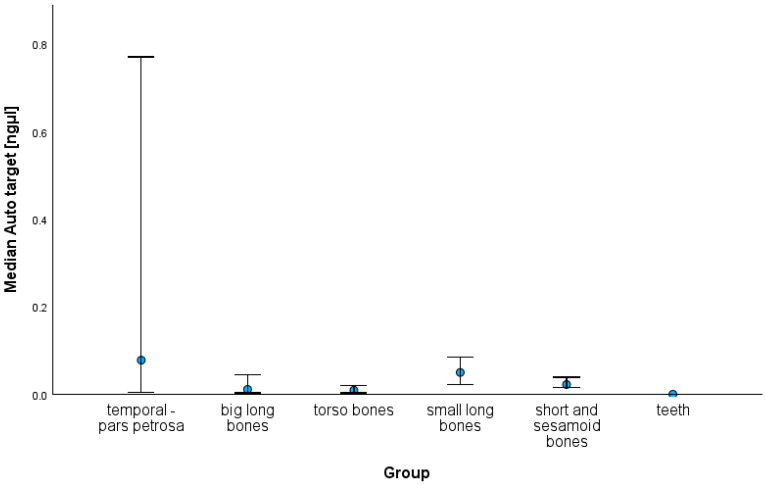
95% confidence intervals for the median for the amount of extracted DNA, by skeletal group, for the Huda Jama database.

To examine differences in DNA yield across different skeletal elements, 95% confidence intervals for medians were calculated using bootstrapping with 10,000 samples (Figure 1).

Analysis of Figure 1 shows statistically significant differences (*p* < 0.05) in DNA concentration between teeth and all other bone categories. The highest median DNA yields were observed in the temporal—pars petrosa group (0.041 < Me < 0.583), followed by small long bones (0.022 < Me < 0.085), short and sesamoid bones (0.016 < Me < 0.037), big long bones (0.005 < Me < 0.015), and torso bones (0.003 < Me < 0.017). Conversely, the teeth group exhibited significantly lower DNA concentrations (0.0001 < Me < 0.0003). No other pairwise comparisons between skeletal groups reached statistical significance at the 0.05 level.

**Figure 2:**
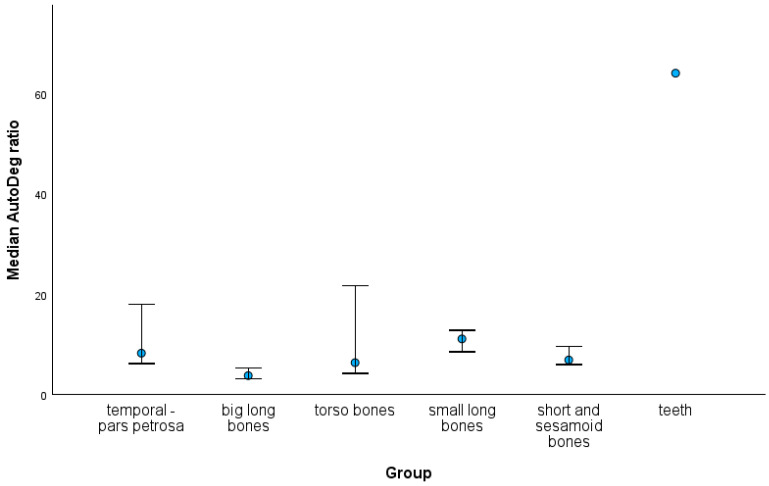
95% confidence intervals for the median for the AutoDeg ratio, by skeletal group, for the Huda Jama database.

Similarly, differences in the Auto/Deg ratio were evaluated using 95% confidence intervals for medians with 10,000 bootstrap samples (Figure 2).

As illustrated in Figure 2, the Auto/Deg ratio for teeth (Me = 64.16) was statistically significantly different (*p* < 0.05) from all other skeletal groups, indicating a distinct degradation pattern. Among the other categories, small long bones showed the highest degradation index (8.52 < Me < 12.81), followed by the temporal—pars petrosa (6.62 < Me < 16.32), short and sesamoid bones (5.95 < Me < 9.57), torso bones (4.25 < Me < 19.82), and big long bones (3.32 < Me < 5.18). No other statistically significant differences in the Auto/Deg ratio were observed between the remaining skeletal pairs.

#### 3.2.2. Results for Konfin II

Descriptive statistics (Table 5) and normality of distribution were assessed for DNA concentration (Auto target [ng DNA/μL]) and the degradation index (Auto/Deg ratio) for the skeletal remains from Konfin II (Table 6).

The highest median for AutoDeg ratio for bones of KonfinII was detected in big long bones (20.34), teeth (18), and temporal—pars petrosa (17.28). In other bone groups, the median AutoDeg ratio was below 10.

Similar to the Huda Jama dataset, the One-Sample Kolmogorov–Smirnov test indicated that both variables significantly deviated from a normal distribution (*p* < 0.001 in both cases). Consequently, non-parametric methods were utilized for further hypothesis testing.

**Figure 3:**
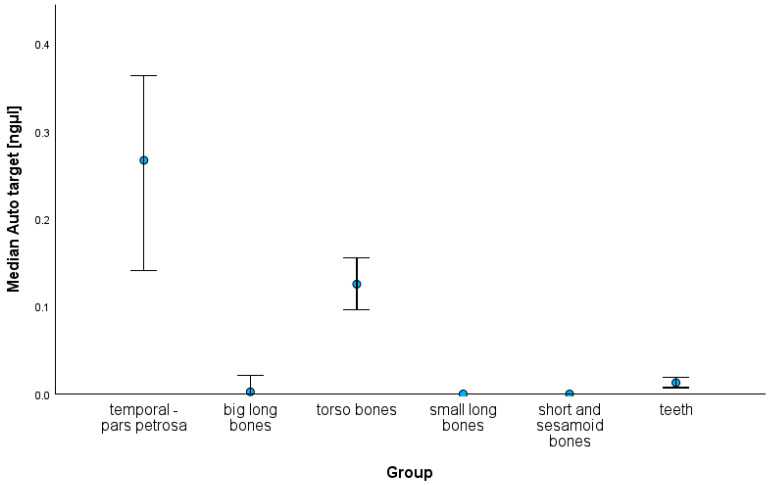
95% confidence intervals for the median for the amount of extracted DNA, by skeletal group, for the Konfin II database.

Differences in DNA yield across the established skeletal groups were evaluated using 95% confidence intervals for medians, calculated with bootstrapping using 10,000 samples (Figure 3).

Analysis of Figure 3 reveals statistically significant differences (*p* < 0.05) in DNA concentration among the various skeletal groups. The temporal—pars petrosa group (0.14 < Me < 0.36) and the torso bones group (0.1 < Me < 0.15) exhibited significantly higher DNA yields compared to big long bones (0.000001 < Me < 0.02), small long bones (Me < 0.000001), and short and sesamoid bones (Me < 0.000001). Additionally, the teeth group showed higher DNA concentrations in comparison to both the small long bones and the short and sesamoid bones groups. No other pairwise comparisons between skeletal groups reached statistical significance at the 0.05 level.

**Figure 4:**
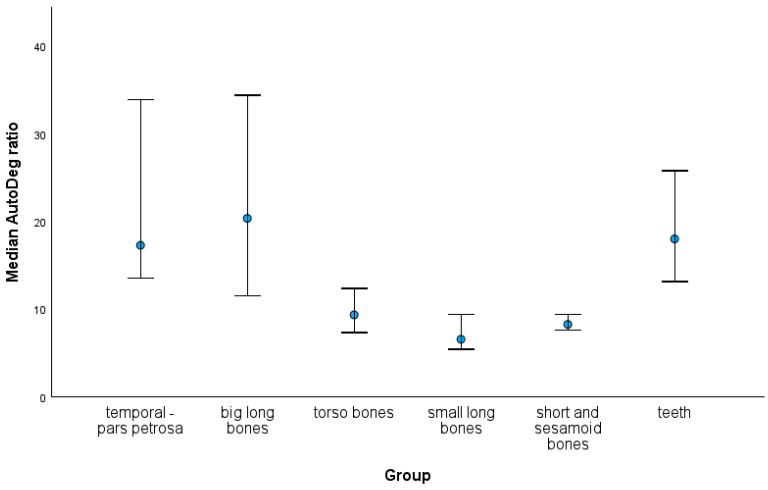
95% confidence intervals for the median for the AutoDeg ratio, by skeletal group, for the Konfin II database.

The Auto/Deg ratio for the Konfin II samples was also analyzed using 95% confidence intervals for medians with 10,000 bootstrap samples (Figure 4).

Statistically significant differences (*p* < 0.05) were observed between several groups. The temporal—pars petrosa (13.53 < Me < 32.55) and teeth (13.15 < Me < 23.67) groups displayed significantly higher degradation indices compared to torso bones (7.66 < Me < 12.2), small long bones (5.59 < Me < 9.32), and short and sesamoid bones (7.64 < Me < 9.39). Furthermore, big long bones (11.71 < Me < 34.38) exhibited a higher Auto/Deg ratio than the small long bones and short and sesamoid bones groups. In all other instances, differences between skeletal pairs were not statistically significant at the 0.05 level.

#### 3.2.3. Comparison Between Samples from Huda Jama and Konfin II

**Figure 5:**
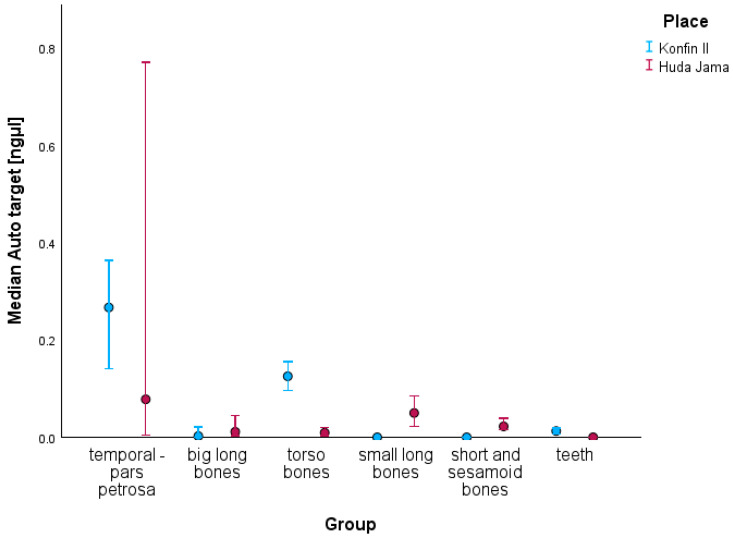
95% confidence intervals for medians for the amount of extracted DNA, by skeletal group.

A comparative analysis of the skeletal remains from Huda Jama and Konfin II was conducted to identify site-specific differences in DNA preservation. Figure 5 illustrates the 95% confidence intervals for medians regarding the DNA yield (Auto target [ng DNA/μL]) across the different skeletal categories for both locations.

Statistical analysis (*p* < 0.05) revealed significant variations in DNA concentration between the two sites depending on the skeletal group. Samples of small long bones from Huda Jama (0.024 < Me < 0.085) yielded significantly higher amounts of DNA than those from Konfin II (Me < 0.000001). A similar trend was observed for short and sesamoid bones, where Huda Jama samples (0.015 < Me < 0.035) outperformed those from Konfin II (Me < 0.000001). Conversely, DNA yields were significantly higher in Konfin II for teeth (0.0071 < Me < 0.0177) and torso bones (0.101 < Me < 0.154) compared to the Huda Jama samples (teeth: 0.0001 < Me < 0.0003; torso bones: 0.003 < Me < 0.017). No statistically significant differences in DNA yield were observed for the remaining bone categories between the two sites.

**Figure 6:**
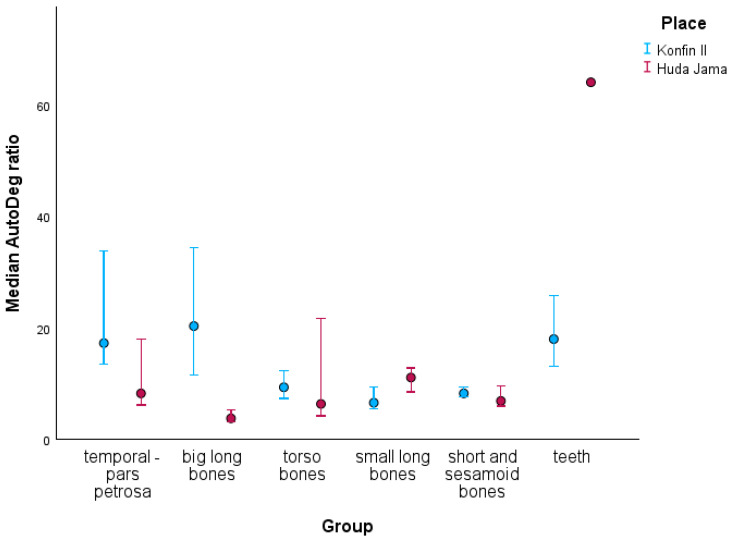
95% confidence intervals for medians for the AutoDeg ratio, by skeletal group.

The DNA degradation patterns were further evaluated by comparing the Auto/Deg ratios, as shown in Figure 6.

Significant differences (*p* < 0.05) in the degradation index were noted for specific skeletal elements. In the big long bones group, samples from Konfin II exhibited a significantly higher Auto/Deg ratio (11.49 < Me < 32.97) compared to those from Huda Jama (3.32 < Me < 5.18). In contrast, the degradation index for teeth was substantially higher in Huda Jama (Me = 64.16) than in Konfin II (13.42 < Me < 25.79). For all other skeletal groups, the differences in the Auto/Deg ratio between the two locations did not reach statistical significance at the 0.05 level.

## 4. Discussion

The results of this study provide a comprehensive insight into the preservation of DNA in aged skeletal remains from two distinct mass grave sites, Huda Jama and Konfin II. By systematically evaluating 560 samples from Konfin II and 225 from Huda Jama, we have demonstrated that DNA recovery is not only dependent on the age of the remains but is heavily influenced by the anatomical type of the bone and the taphonomy of the deposition site [4,5]. Our results indicated significant differences in DNA yield between the Konfin II and Huda Jama sites. While we observed higher DNA concentrations in samples from Huda Jama, it is important to note that the specific taphonomic conditions of each site likely played a role in these outcomes. Due to the historical nature of the sites, precise longitudinal data regarding soil pH, microbial activity, or internal temperature fluctuations were not available. Therefore, this study focuses on the observed differences in DNA preservation across various skeletal elements rather than a direct correlation with specific microenvironmental variables.

Our statistical approach, which utilized non-parametric testing due to the non-normal distribution of DNA yield and degradation metrics, allowed for a robust comparison of six predefined skeletal groups [26,27,28]. The observation that both DNA concentration and the Auto/Deg ratio significantly deviated from normality is consistent with other studies on degraded remains, where taphonomic processes often lead to highly stochastic preservation patterns even within a single skeleton [8,11,15].

One of the most striking findings of this research is the exceptional performance of the petrous part of the temporal bone. In both Huda Jama and Konfin II, the pars petrosa consistently yielded some of the highest DNA concentrations, reinforcing its status as the gold standard in ancient DNA research [13,18]. This is primarily due to the high mineral density of the otic capsule, which provides a superior protective matrix shielding the DNA from microbial infiltration as well as hydrolytic and oxidative damage [3,13]. Our results are consistent with recent comparative studies on aged remains, which further confirm that the petrous bone significantly outperforms other skeletal elements in terms of DNA preservation [9]. In Huda Jama, the median DNA yield for the petrous bone was significantly higher than in teeth, which is a critical observation given that teeth are traditionally favored in forensic identification [7,29]. This trend was even more pronounced in Konfin II, where the petrous bone alongside torso bones outperformed almost all other skeletal elements. These results suggest that when the petrous bone is available, it should be prioritized for genetic analysis, especially in cases where remains have been exposed to challenging taphonomic conditions [9,13]. However, our analysis also revealed a high DNA degradation index for petrous bone samples at both mass grave sites. This paradox—where the temporal—pars petrosa exhibits the highest DNA yield but simultaneously presents an advanced state of fragmentation—carries important implications for the interpretation of genetic data and the suitability of this material. It suggests that while the extreme mineral density of the otic capsule provides an excellent physical barrier that seals the bone and prevents total DNA leaching or complete loss over decades, it does not entirely inhibit internal, spontaneous post-mortem hydrolytic degradation. Consequently, for forensic practice, the pars petrosa remains the highly preferred material because it guarantees the presence of an amplifiable DNA template where other bones fail. If traditional Short Tandem Repeat (STR) typing results in partial profiles, the suitability of the petrous bone can be optimized with advanced downstream technologies (Massively Parallel Sequencing (MPS) or Single Nucleotide Polymorphism (SNP) panels that specifically target shorter DNA fragments).

Although teeth are a common source for DNA, our results showing high fragmentation suggest that dental tissues may be more vulnerable than previously thought. This is further supported by research into different dental tissues, which indicates that nuclear DNA preservation can vary significantly between the pulp, dentin, and cementum [29].

The comparison between the two sites revealed significant differences. While Huda Jama generally showed better DNA preservation in small long bones and short/sesamoid bones, Konfin II exhibited superior preservation in teeth and torso bones. The higher DNA yield in torso bones from Konfin II, particularly ribs and vertebrae, was unexpected given their high proportion of cancellous bone, which is typically more susceptible to degradation [8,12]. The preservation in torso bones might be attributed to the specific positioning of the skeletons or rapid burial, which could have provided localized protection to the spongy bone matrix [15]. Under such conditions, it is also possible that DNA was preserved within the soft tissues remaining inside the spongy bone matrix [12]. Relatively high DNA yield from torso bones in Konfin II challenges the traditional hierarchy of bone sampling, which typically prioritizes long cortical bones. Our results align with previous studies on Slovenian WWII victims, which have indicated that ribs and vertebrae can be reliable sources of genetic material, despite exhibiting intra-bone variability that forensic practitioners must account for [20,21]. Similarly, small bones of the hands and feet, specifically metacarpals and metatarsals, have shown high DNA potential in various Slovenian mass grave contexts. These elements can serve as viable secondary options or even primary targets when the petrous bone is unavailable or when traditional long bones are too degraded [11,18,19]. Together, these findings suggest that the skeletal sampling strategy should be expanded beyond the classical selection of teeth and femora to include both torso and small extremity bones. Data regarding the Auto/Deg ratio provided further depth to our understanding of the state of the remains. A high degradation index signifies that the DNA is heavily fragmented, which can complicate the amplification of longer STR markers [3]. In Huda Jama, teeth were found to be the most degraded elements, with an exceptionally high median Auto/Deg ratio of 64.16. This suggests that while the enamel provides a physical barrier, the internal pulp cavity and dentin may undergo significant biochemical breakdown under the distinct depositional conditions of an enclosed, high-humidity environment like Huda Jama [14]. Conversely, in Konfin II, the big long bones showed a higher degradation ratio compared to Huda Jama, while the teeth were relatively better preserved. This inverse relationship highlights the complexity of taphonomy; no single skeletal type is universally the “best” across different mass grave contexts [8,16]. The higher degradation in big long bones at Konfin II might be related to their larger surface area and the presence of the medullary cavity, which are more exposed to the surrounding matrix compared to other bone types [15]. For all other skeletal groups, including small long bones and short/sesamoid bones, the degradation levels did not differ significantly between the two sites, suggesting that while the total DNA yield was lower in Konfin II for these elements, the quality of the recovered DNA remained comparable.

The high efficiency of the magnetic bead-based purification using the EZ1&2 DNA Investigator kit [23] was evident across the dataset. The fact that only a single sample out of two hundred showed significant inhibition (IPC shift > 0.3) underscores the effectiveness of modern extraction protocols in removing co-extracted humic acids and other PCR inhibitors common in mass grave environments [1]. Technical reliability of qPCR is crucial for forensic laboratories tasked with large-scale identifications, as it minimizes the need for costly and time-consuming re-extractions [2]. The detection limits established for our extraction-negative controls also confirmed that the rigorous anti-contamination measures, including the use of dedicated clean rooms and an elimination database, were successful in maintaining the authenticity of the genetic data [22].

The findings related to small long bones and sesamoid bones also merit discussion. In Huda Jama, these bones performed surprisingly well, whereas in Konfin II, their DNA yields were lower. This suggests that within specific burial contexts characterized by minimal bone movement or rapid sealing, small bones of the hands and feet may serve as viable alternatives when more traditional elements like the femur or teeth are missing or damaged [6]. However, the inconsistency of these bones between the two sites suggests they should be used as secondary options.

The results also highlight the importance of prompt analysis after exhumation. DNA stability is often compromised once remains are removed from their burial context and exposed to unregulated atmospheric conditions. In Huda Jama and Konfin II, the samples were processed shortly after excavation, which may have contributed to the relatively high DNA yields observed in certain skeletal groups. This aligns with findings by Pruvost [30], suggesting that freshly excavated bones are often the best candidates for DNA amplification. If immediate analysis is not possible, maintaining strict control over storage temperature (16–20 °C) and relative humidity (45–65%) is essential to prevent further spontaneous degradation, mold growth, or bone cracking [17]. Furthermore, factors such as UV light exposure must be minimized to prevent additional DNA mutations and density loss in the samples [17].

## 5. Conclusions

This study demonstrates that DNA yield and degradation levels vary significantly both between mass grave sites and among different types of bones. Our results confirm that the petrous part of the temporal bone remains the most reliable and robust source for DNA recovery in WWII mass grave contexts, consistently providing higher yields compared to other skeletal elements. However, the high degradation index observed in these samples suggests that while the dense mineral matrix shields DNA from total loss, the preserved genetic material is often highly fragmented, which may necessitate advanced downstream applications beyond traditional STR typing.

Furthermore, the findings challenge the traditional sampling hierarchy by indicating that torso bones (ribs and vertebrae) and small long bones of the extremities can serve as viable primary or secondary targets for genetic analysis, particularly when their performance is evaluated within the specific taphonomic context of the site. The poor performance of teeth in the Huda Jama environment serves as a critical reminder that relying solely on dental remains may limit identification success in certain mass grave contexts.

While the lack of longitudinal environmental monitoring at the time of excavation limits our ability to correlate DNA quality with precise soil or climatic factors, the comparative data provided here offer valuable insights for forensic practice. We recommend a flexible, site-specific sampling strategy that prioritizes the petrous bone while accounting for the varying preservation levels of other skeletal remains. The integration of qPCR metrics, such as the Auto/Deg ratio, remains essential for predicting the success of STR profiling and for optimizing the selection of skeletal elements in the forensic identification of highly degraded human remains.

## Figures and Tables

**Table 1:** Descriptive statistics for the AutoDeg ratio for the Huda Jama database.

Group	Mean	N	Std. Deviation	Maximum
temporal—pars patrosa	10.230	7	4.830	17.990
big long bones	4.324	14	1.777	8.250
torso bones	9.379	20	7.806	25.910
small long bones	15.042	66	15.675	98.010
short and sesamoid bones	9.953	48	7.801	34.530
teeth	11.370	7	16.557	47.600

**Table 2:** Descriptive statistics for the AutoDeg ratio for the KonfinII database.

Group	Mean	N	Std. Deviation	Maximum
temporal—pars patrosa	25.009	20	16.064	64.850
big long bones	28.031	39	23.2260	100.350
torso bones	12.886	63	12.555	71.180
short and sesamoid bones	10.460	132	8.426	53.930
teeth	32.900	52	79.218	444.730
metatarsals	8.830	116	11.356	78.350
metacarpals	25.745	71	33.661	198.590

**Table 3:** Descriptive statistics for the AutoDeg ratio, for the Huda Jama database.

Group	N	Maximum	Median
temporal—pars petrosa	7	17.990	8.240
big long bones	14	8.250	3.760
torso bones	24	33.716	6.345
small long bones	72	113.685	11.090
short and sesamoid bones	50	42.331	6.885
teeth	34	64.157	64.157

**Table 4:** Test for normality of the distribution of the amount of DNA and the AutoDeg ratio, for the Huda Jama database.

One-Sample Kolmogorov–Smirnov Test				
			Auto target [ng/μL]	AutoDeg ratio
N			201	201
Normal Parameters ^a,b^	Mean		0.062	22.406
	Std. Deviation		0.112	26.558
Most Extreme Differences	Absolute		0.292	0.272
	Positive		0.255	0.272
	Negative		−0.292	−0.218
Test Statistic			0.292	0.272
Asymp. Sig. (2-tailed) ^c^			<0.001	<0.001
Monte Carlo Sig. (2-tailed) ^d^	Sig.		<0.001	<0.001
	99% Confidence Interval	Lower Bound	0.000	0.000
		Upper Bound	0.000	0.000

^a^. Test distribution is Normal. ^b^. Calculated from data. ^c^. Lilliefors Significance Correction. ^d^. Lilliefors’ method based on 10,000 Monte Carlo samples with starting seed 926,214,481.

**Table 5:** Descriptive statistics (after imputation of missing values) for the AutoDeg ratio, for the Konfin II database.

Group	N	Maximum	Median
temporal—pars petrosa	20	64.850	17.275
big long bones	40	123.580	20.340
torso bones	64	83.735	9.340
small long bones	193	222.546	6.560
short and sesamoid bones	141	62.356	8.250
teeth	70	523.948	18.000

**Table 6:** Test for normality of the distribution of the amount of DNA and the AutoDeg ratio, for the Konfin II database.

One-Sample Kolmogorov–Smirnov Test				
			Auto target [ng/μL]	AutoDeg ratio
N			528	528
Normal Parameters ^a,b^	Mean		0.047	37.659
	Std. Deviation		0.138	99.201
Most Extreme Differences	Absolute		0.366	0.358
	Positive		0.332	0.358
	Negative		−0.366	−0.358
Test Statistic			0.366	0.358
Asymp. Sig. (2-tailed) ^c^			<0.001	<0.001
Monte Carlo Sig. (2-tailed) ^d^	Sig.		<0.001	<0.001
	99% Confidence Interval	Lower Bound	0.000	0.000
		Upper Bound	0.000	0.000

^a^. Test distribution is Normal. ^b^. Calculated from data. ^c^. Lilliefors Significance Correction. ^d^. Lilliefors’ method based on 10,000 Monte Carlo samples with starting seed 624,387,341.

## Data Availability

The original contributions presented in this study are included in the article/Supplementary Materials. Further inquiries can be directed to the corresponding author.

## Supplementary Materials

The following supporting information can be downloaded at: https://www.mdpi.com/article/10.3390/genes17060719/s1, Table S1: DNA quantity and degradation parameters of skeletal elements from the Huda Jama mass grave, determined by the PowerQuant analysis (PQ Auto, PQ Deg, and PQ Y in ng/µL, PQ IPC Shift, and PQ Auto/Deg ratio).
